# Thyroid Cancer Incidence in Bulgaria before and after the Introduction of Universal Salt Iodization: An Analysis of the National Cancer Registry Data

**DOI:** 10.4274/balkanmedj.galenos.2020.2019.10.5

**Published:** 2020-10-23

**Authors:** Ludmila Borislavova Ivanova, Mircho Ivanov Vukov, Zdravka Gardeva Vassileva-Valerianova

**Affiliations:** 1 Department of Neurology, Psychiatry, Physiotherapy and Rehabilitation, Preventive Medicine, and Public Health Sofia University “St. Kl. Ohridski” School of Medicine, Sofia, Bulgaria; 2 Statistition-Consultant Bulgarian National Cancer Registry, Sofia, Bulgaria; 3 Clinic of Oncology, Bulgarian National Cancer Registry University Hospital, Sofia, Bulgaria

**Keywords:** Bulgaria, cancer, epidemiology, incidence, thyroid

## Abstract

**Background:**

Thyroid cancer is the most common malignancy of the endocrine system and it has become the fastest growing cancer among women. The suspected risk factors include increased exposure to ionizing radiation during childhood, environmental pollutants, possible iodine deficiency, and excessive iodine exposure.

**Aims:**

To analyze the thyroid cancer incidence between 1980 and 2013 in Bulgaria and to determine the incidence rate before and after the introduction of universal salt iodization in 1994 in regions with different iodine deficiency levels.

**Study Design:**

Retrospective cohort.

**Methods:**

The study was a retrospective analysis of the total number of thyroid cancer cases with all histological types in Bulgaria (thyroid cancer, ICD10 code C73), diagnosed between 01/01/1980 and 31/12/2013, and retrieved from the anonymous cancer registry database of the Bulgarian National Cancer Registry. Age-standardized rates of thyroid cancer per 100,000 persons were calculated for each year of the periods mentioned below by sex and age, utilizing the WHO world reference populations with a special statistical module of the Bulgarian National Cancer Registry’s software CancerRegBG, 2011. Incidence rates were reported by age, sex, and period of diagnosis (1980-86, 1987-93, 1994-99, 2000-2006, 2007-2013). Trends among males and females were analyzed separately, as well as by age category: 0-19, 20-44, 45-64, and 65+. Annual percentage changes of age-standardized incidence rates were analyzed by Joinpoint regression to determine trends using the Joinpoint statistical software SEER* Stat Software, Version 4.1.1, 2014.

**Results:**

The age-standardized rates of thyroid cancer in Bulgaria has been increasing since 1990, being higher among women compared to men (4.68 vs 2.81). The highest age-standardized rates of thyroid cancer was observed in women in the 2007-2013 period. The only significant joinpoint was recorded in 1990 for females and in 1991 for males. The highest incidence rates was in the Smolyan district, a region with historically existing iodine deficiency and relatively high post-Chernobyl radiation exposure.

**Conclusion:**

Our results showed that, in different regions, the age-standardized thyroid cancer rates between endemic and non-endemic differ greatly depending on the radiation dose from the Chernobyl accident. The role of iodine intake in thyroid cancer remains uncertain, but iodine deficiency could be a contributing factor to the increased risk of thyroid cancer.

Thyroid cancer (TC) is the most common malignancy of the endocrine system and it was ranked ninth among women in EU countries in 2012, according to the EUCAN. The estimated incidence of TC in European women was 9.3, threefold more prevalent compared to that in men (1).

Since the end of the last century, TC has become the fastest growing cancer among women, especially in developed countries. In Europe, TC incidence has been rising between 1973-1977 and 1998-2002 for most of the populations, except for Sweden and Norway where the incidence rate (IR) decreased for both males and females (2,3,4).

A significant increase in TC incidence has been observed not only in Europe but also in many other countries around the world during the past several decades (4). In the past three decades, the incidence of TC in the United States has been more than twice higher among individuals with a high socioeconomic status (5). In Canada, since 1970, age-standardized TC IR have increased in women from 3.9-23.4 per 100,000 and in men from 1.5-7.2 per 100,000, while the mortality rates have remained stable (6). Similar trends of increasing TC incidence existed in certain European countries like Italy, Lithuania, Croatia, Denmark, and Finland regardless of the socioeconomic status, diet, and overall morbidity (7).

The reason behind the increased incidence of TC worldwide is still unclear, but there is most likely no single risk factor related to this tendency (8). Improved screening and diagnostic technologies, increased healthcare accessibility and utilization, increased exposure to ionizing radiation especially during childhood, obesity and metabolic syndrome, environmental pollutants, and possibly iodine intake are among the suspected risk factors. The relationship between iodine intake and TC remains unclear, but both iodine deficiency and excessive iodine exposure may contribute to TC, although the extents of these contributions are still not resolved (9,10). Iodine deficiency may induce an increasing incidence of benign thyroid conditions, but high iodine intake also affects thyroid function and possibly TC risk (11). Since “universal iodine supplementation” has been introduced in many countries and the iodine status of the population has improved, it is still under discussion whether the iodine supplementation contributed to increased incidence of cancer, and it has been a topic of controversy and public concern.

Bulgaria is a country with recognized iodine deficiency (12). For the first time, preventive measures to control iodine deficiency were initiated in the late fifties, but they were not sustainable, and in the 1980s, iodine deficiency re-emerged as a public health problem (13). In 1994, universal salt iodization (USI) was implemented on the whole territory of Bulgaria and the country was proclaimed as an “iodine deficiency free country” in 2005 (14). Up to this moment, there were no studies on the incidence of TC before and after the introduction of the USI and the effect of iodine supplementation on the dynamics of TC in regions with different iodine status in Bulgaria.

This study aimed to analyze TC incidence during the 1980-2013 period in Bulgaria and to determine the incidence of TC in Bulgaria before and after the introduction of USI in regions with different levels of iodine deficiency before the introduction of USI.

## MATERIALS AND METHODS

The cancer-related data of the total number of TC patients (ICD10 code C73) in Bulgaria diagnosed with all histological types between 01/01/1980 and 31/12/2013 were retrieved from the anonymous cancer registry database of the Bulgarian National Cancer Registry (BNCR). The BNCR is population-based, and covers the whole country and regularly receives information on new cancer cases (ICD10 codes C00-C96, D00-D09, and D37-D48) from the 13 Regional Cancer Registries (RCRs). The RCRs collect information on cancer patients diagnosed and treated in hospitals, clinics, centers, and other health establishments within their region. Each RCR is responsible for one, two, or three from all 28 administrative districts in the country. In the RCRs, information regarding cancer cases is extracted, coded, and recorded in a specialized registration information system (CancerRegBG), which complies with international classifications and standards. RCRs use two methods of data collection: passive and active, which has been more prevalent in recent years. Each RCR maintains regional databases containing information on registered cases. Regularly, these regional databases are sent to the BNCR to be combined into a national database (15). Data obtained after 2013 were not included in the study, as there is a tendency toward a sharp decline in the number of newly registered cases of cancer (total, by site, gender, age groups, etc), as well as the rough and standardized frequency and mortality, which due to regulatory and financial problems led to the incomplete registration of malignant diseases in the country.

IR per 100,000 persons were reported by age, sex, and period of diagnosis (1980-1986, 1987-1993, 1994-1999, 2000-2006, 2007-2013). Trends among men and women were analyzed separately and by age category, including 0-19, 20-44, 45-64, and 65+.

Age groups were determined based on the number of cases and standardized IR (world standard) in men and women at a 5-year interval. The groups are combined because there are single cases in the age group 0-19 year; the 20-44 group has the highest IR for women and the 45-65 group for men. After 65 years, the IRs declined significantly.

Basic descriptive statistics were performed. Age-standardized rates (ASR) of TC per 100.000 persons were calculated for each year of the periods mentioned below, by sex and age, utilizing the WHO world reference populations (16), and were calculated using a special statistical module of the BNCR’s software CancerRegBG, 2011. Standard errors for the ASRs were calculated as described by Boyle and Parkin (17). Ninety-five percent confidence intervals (95% CI) of IR were estimated using Microsoft Excel version, 2013. The average ASR over each of the five calendar periods (1980-1986, 1987-1993, 1994-1999, 2000-2006, 2007-2013) were calculated using Microsoft Excel version, 2013.

Annual percentage changes of age-standardized IR were analyzed by Joinpoint regression to determine trends using a Joinpoint statistical software SEER* Stat Software, Version 4.1.1,2014 (https://seer.cancer.gov/seerstat/. The software takes trend data (e.g., cancer rates) and fits the simplest Joinpoint model that the data allow and is used for calculation of the cancer trends. Joinpoint regression analysis identifies points where a statistically significant change over time occurred in the linear slope of the trend. Adjacent segments join at points called join points, which indicate statistically significant changes in the time trend (p<0.05) (17). A comparison of the confidence intervals were used to compare the age groups and time categories. When the 95% confidence intervals of the means of two independent populations do not overlap, there is a statistically significant difference between the means (at a significance level of 0.05) (18).

## RESULTS

The incidence of TC in Bulgaria has been increasing since 1990. It almost doubled between 1987-1993 and 2007-2013. The total TC IRs, distinguished by sex from 1980 to 2013 are presented in Table 1. The overall age-adjusted rate (WHO age-standardized rate) increased from 1.74 cases per 100,000 persons in 1994-1999 to 3.05 cases per 100,000 in 2007-2013, with an average APC of 4.20 cases per 100,000 from 1990-2013, being higher among women compared to men (4.68 and 3.55, respectively) (Table 1). The comparison of the ASR before and after 1986 showed a significantly increased IR since 1986 (p<0.05).

**Figure 1:**
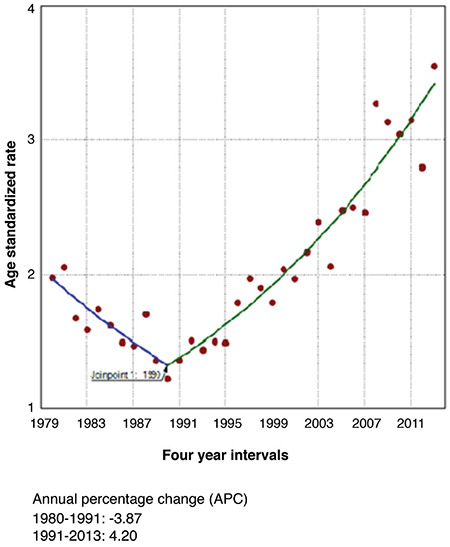
Total incidence of thyroid cancer in Bulgaria using Joinpoint analyses for the period 1980-2013.

**Figure 2:**
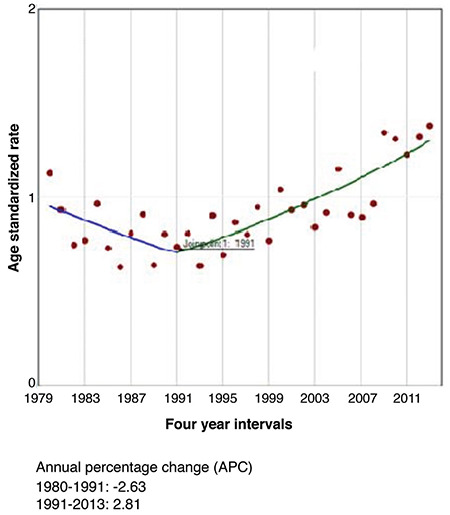
Incidence of thyroid cancer in male individuals in Bulgaria using Joinpoint analyses for the period 1980-2013.

**Figure 3:**
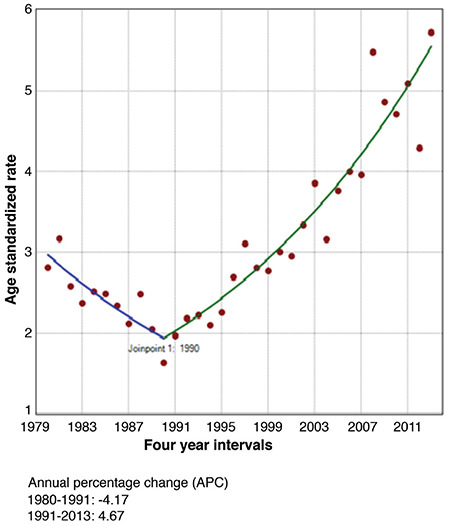
Incidence of thyroid cancer in female individuals in Bulgaria using Joinpoint analyses for the period 1980-2013.

Some differences in the incidence trends over the studied period from 1980-2013 were observed. The only one significant Joinpoint was identified in 1990 for females and in 1991 for males during the entire 1980-2013 period, when the APC (-3.87) from negative over 1980-1990 became steadily growing during the next period of time, from 1990-2013 for both sexes (Figure 1, 2, 3).

The highest ASR of TC was observed in women from 2007-2013 (ASR 4.86, 95% CI: 4.61-5.10) and in men for the same time period (ASR 1.21, 95% CI: 1.09-1.33). During the whole period under analysis, the ASR of women was higher than that of men, three times in 1980-1986 (2.46/0.84 per 100,000, respectively) and four times in 2007 (4.86/1.21 per 100,000, respectively) (Table 1). The increase was statistically significant for each of the monitored time periods both in the whole population and in the female group. The age-adjusted IR of TC was most pronounced in the age group of 20-44 years, especially among women, and the increase was statistically significant compared to the previous 0-19 years age group for all the time intervals. (Table 2).

We compared the total ASR of TC from 1993-2013 in four geographically separated populations, each with a different historical prevalence of iodine deficiency (Pleven and Dobrich, nonendemic and Smolyan and Vratsa, endemic regions) after the introduction of USI in 1994 (Table 3). A significant difference was observed in the incidence of TC between the selected regions, endemic or nonendemic. The total incidence of TC tended to be higher in endemic than in the non-endemic areas. When the results were broken down by sex, this trend was not very obvious.

The region with the highest prevalence of TC was Smolyan (a region with a long history of iodine deficiency), including not only the total rate, but also the rate by male/female, compared to other regions of the country (Table 3). The established prevalence of TC in Smolyan among women was the highest in Bulgaria for the overall observed period since 1993. The endemic region of Vratsa, also with a history of iodine deficiency, ranked second. Although Dobrich and Pleven are known as nonendemic areas, a high incidence of cancer, especially among women, was also registered.

## DISCUSSION

TC is the most common malignant disease of the endocrine system. The IR has steadily increased in developed countries (4,8,19,20). The same trend was observed in Bulgaria since the early 1990s. Bulgaria, as an iodine deficient country, adopted the mandatory salt iodization in 1994, shortly after the Chernobyl accident. During the Chernobyl fallout, the prevention of iodine deficiency was vague and the population was left without any iodine supplementation (13,14). The steady increase of the incidence of TC in Bulgaria in both sexes started in 1990-1991 and is still on-going, showing an increasing trend over different time periods. Joinpoint analysis showed that there was only one point of significant refraction of cancer incidence, where the downward trend began to increase in 1990 (males) and 1991 (females). While in the 1987-1986 period the ASR of the total population was 1.73, it reached 3.05 in 2007-2013, which represented a 2.5-fold increase over a relatively short time. This steady trend was consistent with the overall global trend toward increased TC in recent decades (7,11,21,22).

The upward trend in Bulgaria since 1990 could be attributed, as in many other developed countries, partially to the improved early detection of smaller nodules (<2 cm), better reporting and registration, introduction of more accurate equipment and a broader coverage of the population with ultrasound detection, and fine needle aspiration biopsies (23).

Bulgaria is among the countries with a relatively low incidence of TC in Europe. The average IR of TC in Bulgaria is twice lower than the European average, 3.05 vs 6.3 per 100 000 population in 2012, with large regional differences observed in Europe. The incidence of TC was lower compared to Lithuania (15.5), Italy (13.5), and Austria (12.4), but the positive increasing trend is alarming (1).

As in most countries, TC cancer in Bulgaria occured more frequently in women, approximately three to four times higher than that in men, corresponding to the general trend that the female population is at a higher risk of thyroid diseases including neoplasms (22,24,25). The age trend showed a reversed U-shaped effect, with the highest incidence among women aged 20-44 in 2007-2013. This differed from most data showing that the highest incidence of TC was among women in the 45-60 years age group of (23). Some suspected risk factors for TC included radiation exposure especially during childhood, environmental pollution, history of thyroid diseases, and low or high iodine intake (10). The effect of radiation from the Chernobyl disaster on the iodine deficient youngest population in 1986 could be the reason of the relatively higher ASR in the female 20-44 years age group from Bulgaria (ASR 2.22) compared with the older population groups of 45-64 and 65+ (2.08 and 0.35). We assume that this is the effect of the Chernobyl accident on the iodine deficient children population, which in 1986 was under the age of 10, particularly sensitive to radiation. The much higher effective dose of exposure of thyroid gland to 131Iodine in children compared with adults in Bulgaria was established in 1988, two years after the accident (0.50 vs 0.17 mSv). The greatest exposures were in regions of higher altitudes above sea level, as well as in those where the fallout density was highest (settlements above 0.8 km altitude) and some others on the path of the radioactive cloud. Smolyan and Dobrich were such cities where the highest incidence of TC in both sexes was found (25,26,27). Although a significant part of the territory of Bulgaria was affected by the radiation cloud, the Chernobyl effect on the incidence of cancer in the 1990-2003 period in regions with different radioactive contamination has not been proven (28). Our results on the incidence of TC over a longer period of time, 1993-2013, showed a significantly higher incidence in Smolyan, Dobrich, and Pleven compared to Vratsa, where the total effective dose was the lowest.

The highest incidence of TC among women in Smolyan was probably due to the radiation exposure against a background of long-lasting iodine deficiency, thyroid diseases,s and low environmental selenium availability (28). Although the population of Vratsa had received relatively low radiation dose in 1986, the overall incidence of cancer and that among women was considerable (25). This might be the effect of radiation on an iodine deficient population exposed to industrial environmental pollution.

In Bulgaria, TC IR have increased since 1990 following global trends, but still remain moderate on the European map. The reasons might be improved diagnostics and registration, pre-existing iodine deficiency, and post-Chernobyl radiation. The role of iodine intake in TC remains uncertain, but iodine deficiency could be a contributing factor to the increased risk of TC. The results of our analyses showed that TC in the different regions, endemic and non-endemic, differed depending on the radiation dose during Chernobyl, but the previously existing iodine deficiency may also play a role since it equally increased the risk in areas with relatively low exposure.

The comparison of the total incidence of TC between the endemic and nonendemic regions after the introduction of universal iodization showed that the highest incidence was observed in Smolyan and Vratsa, both regions with a pre-existing deficiency.

Currently, iodine deficiency in Bulgaria is under control because of the universal iodization of salt. More detailed comprehensive studies are needed to identify trends in the incidence and the subtype of TC in a population without iodine deficiency.

## Figures and Tables

**Table 1:**
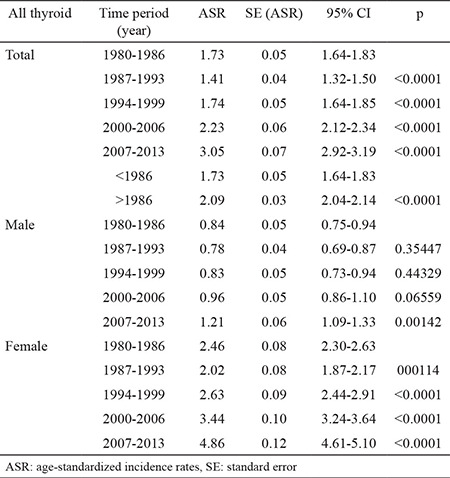
Age-standardized incidence rates with 95% confidence interval (95%) and p-value of thyroid cancer by sex and time periods in Bulgaria using joinpoint analysis for the 1980-2013 period

**Table 2:**
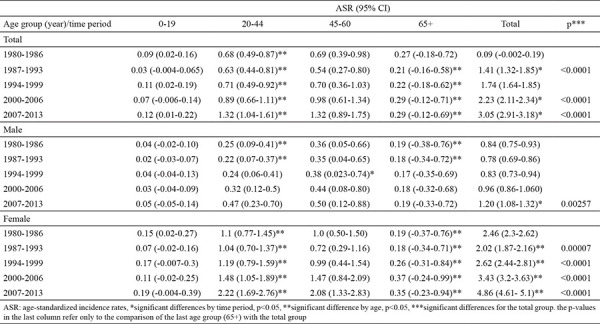
Age-standardized incidence rates (per 100,000, world standard) of thyroid cancer by age groups and time periods in Bulgaria

**Table 3:**
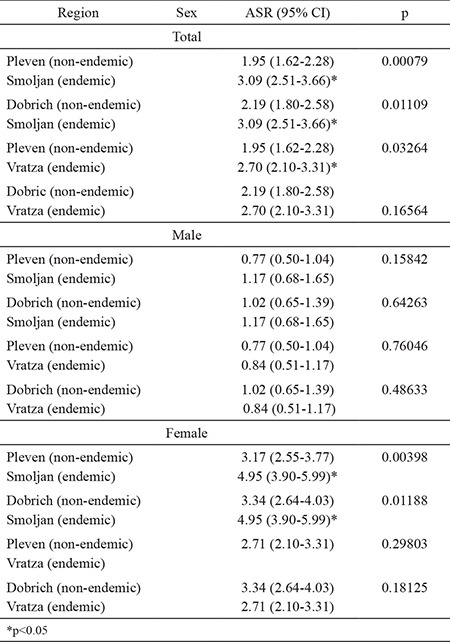
Age-standardized incidence rates with 95% confidence interval of thyroid cancer by sex and endemic/nonendemic regions in Bulgaria for the 1993-2013 period
